# Airway smooth muscle as a target of asthma therapy: history and new directions

**DOI:** 10.1186/1465-9921-7-123

**Published:** 2006-09-29

**Authors:** Luke J Janssen, Kieran Killian

**Affiliations:** 1 Firestone Institute for Respiratory Health, St. Joseph's Hospital and the Department of Medicine, McMaster University, Hamilton, Ontario, L8N 3Z5, Canada

## Abstract

Ultimately, asthma is a disease characterized by constriction of airway smooth muscle (ASM). The earliest approach to the treatment of asthma comprised the use of xanthines and anti-cholinergics with the later introduction of anti-histamines and anti-leukotrienes. Agents directed at ion channels on the smooth muscle membrane (Ca^2+ ^channel blockers, K^+ ^channel openers) have been tried and found to be ineffective. Functional antagonists, which modulate intracellular signalling pathways within the smooth muscle (β-agonists and phosphodiesterase inhibitors), have been used for decades with success, but are not universally effective and patients continue to suffer with exacerbations of asthma using these drugs. During the past several decades, research energies have been directed into developing therapies to treat airway inflammation, but there have been no substantial advances in asthma therapies targeting the ASM. In this manuscript, excitation-contraction coupling in ASM is addressed, highlighting the current treatment of asthma while proposing several new directions that may prove helpful in the management of this disease.

## Background

Asthma is experienced during the life span of approximately 10% of the population, resulting in morbidity and mortality costing a substantial economic burden on society [1]. The predominant feature of asthma is the discomfort experienced upon breathing in the presence of excessive and inappropriate constriction of the airway smooth muscle (ASM). Although airway inflammation may play an important role in asthma, it is benign in the absence of airway narrowing. The patient is thus predominantly concerned with narrowing of their airways, contributing to an unpleasant increase in the effort required to breathe; in the extreme, this increased effort fails to allow sufficient ventilation, leading to morbidity and even mortality. As such, ASM is ultimately a major target in any management of asthma.

The earliest recorded treatments of asthma included tobacco, indian hemp, sedation (using low doses of chloroform, ether, or opium), ipecacuana, coffee, tea, stramonium lobelia and other less effective agents. These agents express the pharmacological properties of the xanthines, cholinergic blockade, sympathetic stimulation, sedation and direct smooth muscle relaxation. Direct approaches using anti-cholinergics, anti-histamines, anti-leukotrienes, and functional antagonists modulating intracellular signalling pathways (β-agonists and phosphodiesterase inhibitors) followed (section 3.2). These have been used for decades with reasonable success, but patients continue to suffer exacerbations of asthma. Research energies were poured into developing new therapies to treat airway inflammation to prevent rather than treat the active disease. Asthma therapies using immune modulation and anti-inflammatory therapies proved to be so successful that targeting the ASM receded. Better understanding of the mechanisms underlying contraction of ASM is still essential to the management of the active disease. In this manuscript, basic excitation-contraction coupling in ASM is summarized and several new directions to the treatment of abnormal smooth muscle constriction are introduced.

### Overview of excitation-contraction coupling

**Figure 1:**
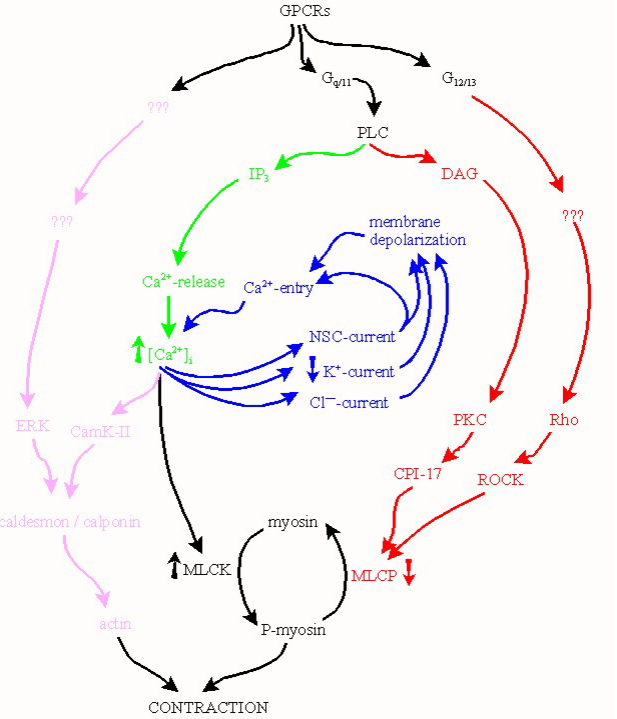
**Bronchoconstrictors act on G-protein coupled receptors coupled to a variety of signalling pathways **involving membrane depolarization (blue), release of internal Ca^2+ ^(green), changes in Ca^2+^-sensitivity (red), and/or thin filament-mediated mechanisms (magenta).

Asthma is characterized by excess reversible constriction and airway hyperresponsiveness (AHR) to a wide variety of spasmogens. Thus, it is essential to understand the mechanisms underlying excitation-contraction coupling of ASM. Contraction is triggered by phosphorylation of myosin. This is catalyzed by Ca^2+^/calmodulin-dependent myosin light chain kinase (MLCK), which in turn is activated as [Ca^2+^]_i _is elevated (see Fig. 1). Mechanisms intrinsic to the thin filament and Ca^2+^-sensitivity are also involved and have the potential for therapeutic intervention in modulating these basic responses.

### Voltage-dependent mechanisms

Excitation-contraction coupling in cardiac, skeletal, vascular and gastrointestinal smooth muscles depends on membrane depolarization resulting in Ca^2+^-entry via voltage-dependent ('L-type') Ca^2+^-channels. As such, Ca^2+^-channel blockers and K^+^-channel openers are invaluable in controlling cardiac and smooth muscle contractions in hypertension, stroke, myocardial infarction, gastrointestinal motility disorders, *etc*. [2-4]. Excitation of ASM is also accompanied by membrane depolarization mediated primarily by Ca^2+^-dependent Cl^--^- and non-selective cation-channels, as well as activation of large voltage-dependent Ca^2+^-currents. The latter can be sufficient to produce contraction, as indicated by the robust responses evoked by potassium chloride or K^+^-channel blockers. As such, a natural conclusion would be that Ca^2+^-channel blockers should be useful in the treatment of asthma: however, they are essentially useless in this respect (see section 9.2).

### Release of internal Ca^2+^

Internally sequestered Ca^2+ ^plays an important role in agonist-evoked responses in ASM. The sarcoplasmic reticulum (SR) is central to this, acting as a sink to buffer cytosolic [Ca^2+^]_i_, as well as providing an agonist-releasable store of Ca^2+ ^to trigger contractions. Most, if not all, bronchoconstrictor autacoids act through G-protein-coupled receptors to stimulate phospholipase C activity and subsequent generation of IP_3_, which in turn signals the SR to release stored Ca^2+ ^(Fig. 1). The mechanisms underlying IP_3_- and ryanodine receptor-mediated release of internal Ca^2+ ^and re-uptake of Ca^2+ ^by the **S**arcoplasmic/**E**ndoplasmic **R**eticulum **C**a^2+^-**A**TPase (SERCA) are well understood, although their relative roles in excitation-contraction coupling may not be. Other aspects of Ca^2+^-handling are very poorly understood, including the mechanism(s) by which the SR is refilled. Greater magnitude of release of Ca^2+ ^in cells/tissues pretreated with allergen or pro-inflammatory cytokines has been documented [5-7]. However, there is little correlation between the magnitude of the initial Ca^2+^-spike, which lasts only a few seconds, and the subsequent contractile response which lasts many minutes or hours. Other groups [8-13] are now focussing their attention on the frequency of repetitive Ca^2+^-spikes following agonist stimulation.

### Changes in Ca^2+^-sensitivity

ASM cells also possess a myosin light chain phosphatase (MLCP) which dephosphorylates myosin, limiting or reversing airway contraction (see Fig. 1). If MLCP activity is down-regulated, net myosin phosphorylation in response to a given change in [Ca^2+^]_i _will be enhanced and/or prolonged, resulting in greater contraction: in other words, the Ca^2+^-sensitivity of the contractile apparatus is increased. At least two different signalling pathways have been found to mediate increased Ca^2+^-sensitivity in ASM, the first involving diacylglycerol (another second messenger liberated by phospholipase C) and protein kinase C: the latter can phosphorylate CPI-17, which regulates MLCP activity.

**Figure 2:**
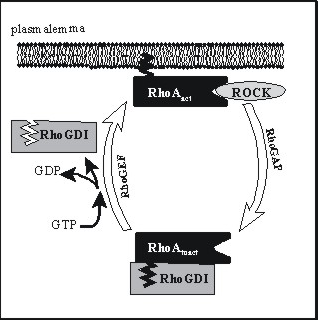
Summary of Rho/ROCK signalling cascade.

The second pathway involves the monomeric G-protein RhoA and its downstream effector molecule Rho-kinase (ROCK). A decade of study in vascular smooth muscle has revealed certain aspects of this signalling cascade (Fig. 2). Inactive RhoA exists in the cytosol with its prenylated hydrophobic tail inserted into its partner molecule, GDP dissociation inhibitor (RhoGDI). G-protein-coupled receptors, upon binding their respective ligands, activate the heterotrimeric G-protein G_12,13_, which in turn triggers one or more tyrosine kinases (c-Src, FAK, Fyn, *etc*.) and other signalling molecules, culminating in the activation of a Rho-specific guanine nucleotide exchange factor (RhoGEF). Numerous GEFs have been identified in the human genome, but the ones most studied include LARG, PDZ-RhoGEF and p115 RhoGEF. These displace RhoGDI and stimulate exchange of GDP for GTP, activating RhoA, which translocates to the membrane and interacts with ROCK. The latter in turn phosphorylates MLCP at two different threonine residues [14] – Thr696 (inhibiting its phosphatase activity) and Thr853 (interfering with its targeting of myosin) – ultimately leading to suppression of MLCP activity. RhoA inactivates by hydrolyzing the GTP bound to it (catalyzed by Rho-GTPase activating protein, or RhoGAP) and re-associating with RhoGDI.

Much of the data summarized above were derived from vascular smooth muscle, which may not be applicable to ASM. There are many examples of how these two tissue types can operate quite differently. For example, the two differ dramatically with respect to the role of Ca^2+^/calmodulin-dependent protein kinase II in activation of RhoA [15]. Likewise, both airway and vascular smooth muscle have exactly the same cellular machinery for voltage-dependent contractions, but have diametrically opposite dependence upon that pathway. Very little is known about the regulation of the Rho/ROCK signalling pathway in ASM, but its exploration may provide novel targets for therapeutic intervention.

### Thin filament-mediated mechanisms

All of the signalling mechanisms summarized above are directed in one way or another at phosphorylation/dephosphorylation of myosin (*i.e*., the "thick filament"). Emerging data now also point to a number of mechanisms pertaining specifically to actin (the "thin filament") [16]. In particular, calponin and caldesmon both interact with F-actin and myosin and inhibit actomyosin ATPase activity. Both are regulated by adrenoceptor-stimulated PKC- and ERK-activities: the latter mediate changes in the phosphorylation state and/or localization of caldesmon and calponin, leading to removal of inhibition of actin, resulting in contraction.

### Evolution of asthma therapy

By and large, the advances made in our understanding of excitation-contraction coupling in ASM have been driven largely from other fields, first in skeletal muscle and later vascular smooth muscle, neither of which are good models for ASM physiology (since their physiology is quite different from that of ASM).

### Basic pharmacology of excitation of ASM

Knowledge of the innervations of the airway and the response of ASM to circulating hormones initiated current therapies. The excitatory innervation of ASM is parasympathetic, exerting its actions primarily through muscarinic cholinergic receptors [17;18]. Cholinergic receptor blockers progressed from belladonna and stramonium lobeline, leading eventually to atropine. Atropine had substantial side effects, given its pleiotropic effects throughout the body. The inhaled route was exploited to direct treatment to the airway but absorption into the circulation led to distal side effects. Ipratropium bromide, not readily absorbed into the bloodstream, eliminated the major side effects, and is an effective bronchodilator. Anti-cholinergics, including ipratropium and its long-acting equivalent tiotropium, have been used to treat asthma but in general adrenergic agents are preferred. Anti-cholinergic agents are used with acute severe asthma but are not broadly used in the day-to-day management of mild to moderate asthma. More selective drugs may prove more useful (*e.g*., M_2_- and M_3_-selective blockers).

Sympathetic stimulation relaxes ASM. The finding that the effects of sympathetic stimulation were mimicked by adrenalin (discovered at the turn of the last millenium) and noradrenalin led to the discovery of chemical neurotransmission. In the 1940's the concept of adrenergic receptor subtypes arose due to the different effects of adrenalin on different tissues. This ultimately led to the discovery of specific agonists causing ASM relaxation (β_2_-receptor agonists). Short- and long-acting β-agonists are now the most widely used bronchodilating agents.

The airways of some species including man exhibit a non-adrenergic, non-cholinergic innervation which make a minor contribution to ASM activity. The agonist for this system is still debated, but may include nitric oxide. As such, nitric oxide may provide a useful target for the treatment of asthma.

Asthma precipitated by allergen exposure in sensitized subjects provides a useful experimental model. Allergen binds to IgE on the surface of mast cells following inhalation leading to the immediate release of histamine, which in turn causes an immediate ("early") bronchoconstrictor response within 10 minutes and lasting approximately 90 minutes. Histamine acts on H_1 _receptors on the ASM, which in turn are coupled to the same signalling pathways utilized by muscarinic receptors (namely, activation of the phosphoinositide cascade, release of internally sequestered Ca^2+ ^and possibly Rho/ROCK-mediated enhancement of Ca^2+^-sensitivity). Anti-histamines have been proven to be partially effective in the treatment of asthma [18].

The early response is followed 6–8 hours later by a second more prolonged bronchoconstriction lasting many hours or even days, mediated in part by a "slow-reacting substance of anaphylaxis", or SRSA [19]. Upon further investigation, leukotrienes proved to be the mysterious SRSA, leading to the award of a Nobel Prize [94]. In addition to their actions on various inflammatory cells (largely mediated by LTB_4_), leukotrienes act on cys-LT_1 _receptors on the ASM: the latter are also G-protein-coupled receptors and, once again, act through stimulation of the phosphoinositide signalling cascade and of the Rho/ROCK-mediated change in Ca^2+^-sensitivity. This led to the development of blockers of those receptors and of leukotriene synthesis (lipoxygenase inhibitors). The efficacy of these agents in the treatment of asthma has been less than that initially expected but these compounds are widely used.

### Functional antagonism of a "convergent signalling pathway" in ASM

**Figure 3:**
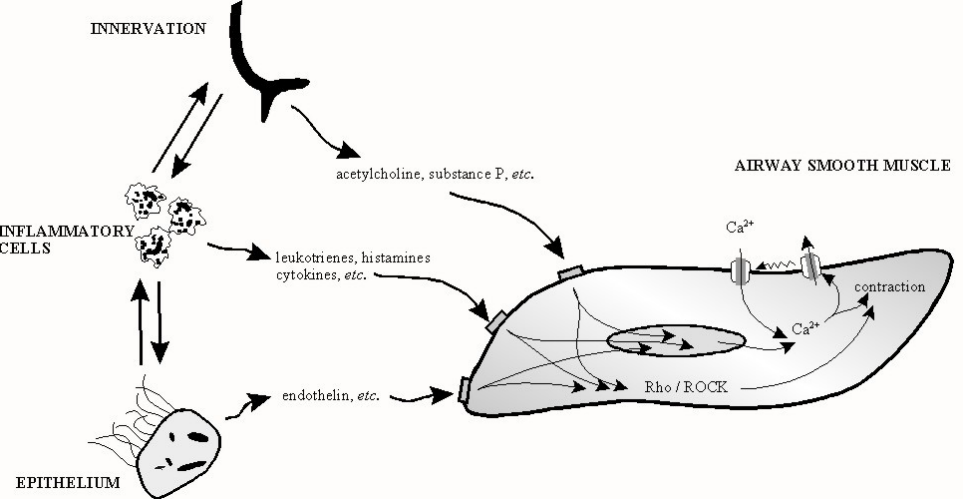
**The non-specific nature of airway hyperreactivity and a convergent signalling pathway for spasmogens: hope for a novel therapy for asthma? **ASM receives diverse excitatory inputs from the innervation, inflammatory cells, and the epithelium, all of which act through distinct receptors, but a common signalling pathway. In asthma, the smooth muscle exhibits increased sensitivity to a wide range of excitatory stimuli. The non-specific nature of airway hyperreactivity suggests that some post-receptor mechanism(s) within the smooth muscle *per se *is altered. Spasmogens act through a convergent signalling pathway involving Ca^2+^-handling and RhoA.

Ironically, the disappointing results of the therapeutic strategies summarized above appear to be due in part to the exceptional pharmacological selectivity of the agents being used. The airways receive numerous excitatory inputs, each acting exclusively on its own distinct plasmalemmal receptor (Fig. 3), and asthma is accompanied by non-specific AHR to a wide variety of excitatory stimuli. As such, an approach which interrupts the intracellular signalling pathways used by many/all of the excitatory stimuli is an exciting prospect.

It was hoped that one such common pathway was voltage-dependent Ca^2+^-influx. The latter is of central importance in cardiac, skeletal, vascular and gastrointestinal muscles, and Ca^2+^-channel blockers are highly useful in many diseases of those tissues [20,21]. There are many lines of evidence which suggest voltage-dependent Ca^2+^-influx should also be important in ASM, including the depolarizing influence of bronchoconstrictors, the hyperpolarizing influence of bronchodilators, the abundance of the very same type of Ca^2+^-channel as is present in the non-airway muscles listed above, and the substantial contractions evoked in ASM by high millimolar potassium chloride. It was natural, then, to believe that asthma might also be treated using Ca^2+^-channel blockers: however, this approach has proven to be useless [22-28]. Despite this setback, others went on to test the potential efficacy of K^+^-channel openers in the treatment of asthma, even though the underlying rationale for such an approach is identical to that of using Ca^2+^-channel blockers (*i.e*, to hyperpolarize the membrane such that Ca^2+^-channels are deactivated). Not surprisingly, this approach was also found to be completely ineffective [29-32]. These and many other findings accumulated over decades of research are most simply interpreted as indicating that voltage-dependent Ca^2+^-influx is not centrally important in ASM contraction and asthma. Nonetheless, even today there still appears to be a tacit adherence to the dogma that such electromechanical coupling is important. A better understanding of contraction/relaxation in ASM demands a new emphasis on mechanisms which are independent of membrane potential (see below).

Another major line of research focussed on those stimuli which exert an inhibitory (*i.e*., relaxant) influence on the ASM. The predominant inhibitory innervation is adrenergic in nature, with the neurotransmitter norepinephrine and circulating catecholamines (particularly epinephrine) acting on β-adrenoceptors (more specifically β_2_-subtype in human and many other species). Binding of these ligands to the β_2_-receptors leads to stimulation of adenylate cyclase, production of cAMP and consequent increase in protein kinase A activity, which in turn mediates many changes that are opposite to those exerted by the bronchoconstrictor agents: vis-a-vis, decreased cytosolic levels of Ca^2+ ^(through a variety of actions on plasmalemmal K^+^- and Ca^2+^-channels [33], as well as the Ca^2+^-pumps on the plasmalemma and the SR [34]), inhibition of the RhoA/ROCK signalling pathway [35] and direct stimulation of MLCP [34]. A more recent development which builds on the knowledge of the actions of cAMP on ASM has been the application of phosphodiesterase inhibitors in the treatment of asthma. These suppress the hydrolysis of cAMP, allowing greater and more prolonged actions upon adrenergic stimulation.

### Anti-inflammatory agents

Given that many of the manifestations of asthma are triggered directly or indirectly by inflammation, asthma treatment is closely allied with immunology. The strategy of interfering with the inflammatory response using an ever longer list of corticosteroids, inhibitors of leukotriene synthesis or leukotriene receptors, blockers of IgE receptors, or of cytokines has been undoubtedly successful. The past decade or two has witnessed a massive research effort to better understand the inflammatory response, with immense resources and energies being directed at identifying newer anti-inflammatory agents. A full description of this body of research is beyond the scope of this communication. Prevention of asthma through these strategies is important but treatment of the acute bronchoconstriction will always be required. "If airway inflammation didn't cause acute bronchoconstriction, asthma might be a more tolerable disease" [36]. The most effective strategy to acutely dilate an airway will always be predicated on understanding the process of excitation-contraction coupling (above) and exploiting those mechanisms. An increasingly familiar experience is inadequate treatment of the airflow limitation associated with asthma.

### Novel directions

Despite all the advances summarized above, and the pharmacological interventions which have arisen from them, it still remains that asthma is not well controlled in many individuals. Clearly, different approaches need to be developed. Acetylcholine, histamine and leukotrienes all act through a convergent signalling pathway (Fig. 3): the same is true for other spasmogens such as endothelin, serotonin, substance P, *etc*.. Appreciation of this fact allows for several potential novel targets to be explored.

### Release of internal Ca^2+^

All of the bronchoconstrictor stimuli referred to above act through G-protein-coupled receptors to stimulate Ca^2+^-release. In contrast to the relative impotence of blockers of voltage-dependent Ca^2+^-influx, a long and ever-growing list of *in vitro *studies of isolated airway tissues attests to the much greater effect of inhibiting IP_3_-induced Ca^2+^-release or of depleting the SR using blockers of SERCA. A major drawback is that this Ca^2+^-homeostatic pathway is central in nearly every cell type in the body, and therefore seems to fail to offer a sufficiently selective target. However, the same criticism can be levelled at many of the other therapeutic approaches which have already been tried (*e.g*., targeting cAMP). It may be possible to identify components of the Ca^2+^-homeostatic pathway which are specific to ASM, and/or to limit delivery of agents by having patients inhale modulators of this pathway.

Recently, a great deal of attention has been focussed on the mechanisms underlying refilling of the SR. In many cells, depletion of the internal Ca^2+^-store triggers a Ca^2+^-influx pathway. We have begun to characterize a membrane current which is evoked in ASM by depletion of the SR using the SERCA inhibitor cyclopiazonic acid [37]. This current exhibits many electrophysiological and pharmacological properties in common with Ca^2+ ^store depletion-activated currents in other cell types referred to as TRP (**T**ransient **R**eceptor **P**otential) currents [38,39]. Surprisingly, several recent reviews [38,40,41] have highlighted the potential of TRP-channels as therapeutic targets in ASM, despite the fact that there had not yet been any direct electrophysiological data pertaining to TRP currents in ASM: up to that point, the supporting data for these currents had been obtained exclusively from studies using fluorimetric Ca^2+^-dyes (which poorly discriminate Ca^2+^-influx pathways) or very indirect approaches based on mechanical responses as indices of Ca^2+^-handling. In both cases, the studies have relied on the dubious selectivities of a variety of pharmacological tools.

Several groups including our own have published data which suggest voltage-dependent Ca^2+^-channels may also contribute to refilling and maintenance of the SR [42-47]. More surprisingly, our data suggest that this refilling pathway in ASM does not involve SERCA, but some novel interaction of the SR and the plasmalemma which allows Ca^2+ ^to flow directly from the extracellular space into the SR [42,43]. Elsewhere, a model has been proposed which describes one such interaction [48-53]. Briefly, agonist-induced depletion of the internal store triggers activation of protein tyrosine kinases and Ras: these cause the cytoskeleton to re-organize in such a way as to directly couple IP_3_-receptors on the SR with Ca^2+^-channels on the plasmalemma. Several observations made in ASM are consistent with such a mechanism: (*i*) spasmogenic stimulation of ASM is accompanied by activation of tyrosine kinases [54-56] and Ras/Rho [57-60], as well as cytoskeletal rearrangement [55,59-61]; (*ii*) tyrosine kinase inhibition compromises SR refilling [62]; (*iii*) ASM depleted of FAK (which regulates cytoskeleton stability) shows marked suppression of cholinergic Ca^2+^-transients and contractions as well as changes in voltage-dependent Ca^2+^-channel function, without any disruptive changes in the contractile apparatus *per se *(assessed by addition of Ca^2+ ^to permeabilized strips) [63]. However, the possible role for this novel SR refilling pathway has not yet been tested in ASM: its presence and operation in ASM would supply another potential target for the treatment of asthma.

Other groups are calling attention to the temporal dynamics of Ca^2+^-signalling rather than merely the amplitude of the Ca^2+^-responses. That is, they show that excitatory stimuli do not simply trigger a solitary rise and fall of [Ca^2+^]_i_, but rather a series of repetitive Ca^2+ ^"spikes" or "waves". More importantly, their data indicate that the strength of the contractile response evoked by a bronchoconstrictor depends not so much on the absolute peak magnitude of the Ca^2+^-elevation, but rather the frequency of the Ca^2+ ^waves [64,65]. As such, it may soon prove possible to modulate airway constriction using agents which modulate Ca^2+^-wave frequency. That is, rather than merely blocking the channels which release internally sequestered Ca^2+ ^from the SR, it may be possible to modulate the kinetics of their activation, thereby affecting the onset of each Ca^2+^-spike. Alternatively, the cellular effectors which determine the decay or resolution of each Ca^2+^-spike may offer useful targets: these include the Ca^2+^-release channels themselves (perhaps it might be possible to accelerate their deactivation or inactivation), as well as the cellular entities which restore [Ca^2+^]_i _to resting levels (the plasmalemmal Ca^2+^-pump, SERCA and Na^+^/Ca^2+ ^exchange).

### Cl^--^-channels

ASM exhibits large Cl^-- ^currents in response to excitatory stimuli, and these are tightly regulated by second messenger signalling events [66-72]. It is usually concluded that Cl^-- ^currents are important for excitation-contraction coupling by depolarizing the membrane and thus triggering voltage-dependent Ca^2+^-influx, and would for this reason provide a potential target for asthma therapy. However, this therapeutic approach should be no more effective than suppressing voltage-dependent Ca^2+^-influx using Ca^2+^-channel blockers or K^+^-channel openers (neither of which have proven to be effective). Why, then, are Cl^--^-channels so prominent in ASM?

A Cl^--^-channel has been isolated from ASM with properties similar to those on the SR of skeletal and cardiac muscle where they facilitate Ca^2+^-flux by neutralizing charge build-up on the SR membranes [73]. We have therefore proposed an entirely novel and testable hypothesis [74]: that agonists activate Cl^-- ^currents in ASM in order to facilitate Ca^2+^-release/uptake. That is, Ca^2+^-efflux from the SR leads to a net negative charge on the inner face of the SR membrane which hinders Ca^2+^-release unless alleviated by compensatory fluxes of Cl^-- ^out of the SR. Given that the agonists trigger substantial plasmalemmal Cl^-- ^currents, the sudden loss of Cl^-- ^from the subplasmalemmal space would instantaneously alter the equilibrium potential for Cl^-- ^across the SR membrane, thereby facilitating efflux of Cl^-- ^(and Ca^2+^) from the SR. Consistent with this, we found contractions evoked by various stimuli including caffeine to be reduced by removing external Cl^-- ^[75]; interestingly, reintroduction of Cl^-- ^restored the initial peak response, suggesting normal refilling of the SR.

Cytosolic [Cl^--^] may also modulate RhoA/ROCK signalling in ASM. While characterizing the agonist-evoked Cl^--^-currents in canine ASM, we noted contractions could be evoked repeatedly during voltage clamp at -60 mV (at which voltage-dependent Ca^2+^-channels are not open) and in the presence of cyclopiazonic acid [43]: such contractions are clearly independent of both voltage-dependent Ca^2+^-influx and release of internal Ca^2+ ^and therefore likely involve altered Ca^2+^-sensitivity of the contractile apparatus. More importantly, we found that cells which were perfused internally with a Cl^--^-deficient electrode solution quickly lost the ability to contract [70]. One interpretation of these findings is that Cl^-- ^is somehow essential to Rho and/or ROCK activation. Consistent with that, we have found that the Cl^--^-channel blocker niflumic acid markedly suppresses cholinergically-induced RhoA-activation. Changes in subplasmalemmal [Cl^--^] might facilitate translocation of RhoA to the membrane, or enhance interactions between the different components of this signalling cascade. Others have shown G-protein activity to be modulated by [Cl^--^] [76]. Alternatively, it might be possible that changes in cytosolic [Cl^--^] somehow affect ROCK activation and/or kinetics.

#### RhoA/ROCK signalling

An ever growing literature attests to the importance of the RhoA/ROCK signalling pathway in increased Ca^2+^-sensitivity of smooth muscle in general. ROCK inhibitors are effective as bronchodilators [35,77-79]. Increased RhoA/ROCK activities have been documented in allergic models of asthma [80-86]. However, little is known about the details underlying activation and modulation of this signalling pathway in ASM. Work done in vascular smooth muscle, or even non-muscle preparations, may not be equally applicable in ASM, as exemplified in the great deal of time and effort spent, and lost, on studying voltage-dependent Ca^2+^-influx in ASM. Also, although many have examined stimulation of the RhoA/ROCK signalling pathway by excitatory agonists [77,79,87-90], very few have looked at the effects of relaxant agonists on this pathway. Recently, we were the first to measure directly the activities of RhoA and ROCK in ASM using immunoprecipitation pull-down and radiometric enzyme assays [15,35,88], and so documented the kinetics of activation of these two signalling molecules: RhoA becomes activated within seconds, reaching a peak within 2 minutes, but then falls back toward baseline even though tone continues to build. We also described the inhibitory effects, particularly on ROCK activity, of two different β-agonists – isoproterenol (a short-acting, non-selective β-agonist with full agonist activity) and salmeterol (a long-acting, β_2_-selective agonist with only partial agonist activity), both of which signal through stimulation of adenylate cyclase activity – and a nitric oxide donor (S-nitroso-N-acetylpenicillamine; acting through stimulation of guanylate cyclase).

Many of the details underlying RhoA/ROCK activation remain to be explored. We were the first to show in ASM that RhoA is activated by potassium chloride [88]. Follow up work showed that this is directly related to elevated [Ca^2+^]_i_, although membrane depolarization *per se *may also be involved. Changes in ROCK activity parallelled those in RhoA, suggesting KCl is not exerting an additional effect on ROCK (*i.e*., is only stimulating RhoA). How might Ca^2+ ^and membrane voltage stimulate RhoA activity? It may be that Rho-activation is Ca^2+^-dependent, although this explanation must explain the relative inefficacy of Ca^2+^-channel blockers. Alternatively, proteins are charged molecules, and those which need to translocate to the membrane must by influenced by the transmembrane voltage gradient. On the other hand, there is a growing literature describing direct physical interactions between various enzymes and ion channels, including "L-type" Ca^2+^-channels [91,92]. It is possible that depolarization-induced conformational changes in the channel proteins are transduced to accessory cytosolic proteins including RhoA; Ca^2+^-channel blockers do not necessarily affect those conformational changes, which could explain why ASM is refractory to that class of drug. None of these possibilities have been explored sufficiently.

Finally, the downstream targets which ROCK must phosphorylate to evoke contraction have not been examined in detail. MLCP may be the primary target [93]. However, data from non-airway tissues suggest that ROCK also phosphorylates myosin light chain *per se *[94,95], ezrin/radixin/moesin family proteins [96-99], elongation factor-1α [100], adducin [99,101], intermediate filaments [102-104], and LIM-kinase [105,106]. There likely are other targets which have not yet been revealed.

HMG-CoA reductase inhibitors or 'statins' are widely used to normalize hypercholesterolemia [107]. However, it is now becoming clear that their beneficial effects may not only lie in their ability to decrease cholesterol synthesis *per se *[108]. Geranylgeranylpyrophosphate, an isoprenoid intermediate arising from this biosynthetic pathway, is essential in the activation of RhoA. As such, statins may also act by suppressing Rho/ROCK signalling, a pharmacological action which might be exploited in asthma.

### Tyrosine kinase(s)

We have shown the non-specific tyrosine kinase inhibitor genistein to have powerful inhibitory effects on cholinergic responses in ASM [89]. However, the identity of the tyrosine kinase(s) and the target(s) of its stimulation are largely unclear. There is currently a great deal of attention being focussed upon the role(s) of FAK in cholinergic responses in ASM [109-111]: upon stimulation, FAK can be autophosphorylated on tyrosine 397, recruiting other non-receptor PTKs such as pp60^src ^and pp59^fyn ^(via their SH2 domains), which can create additional tyrosine phosphorylation on other residues of FAK. Also, there is reason to believe that tyrosine phosphorylation is part of the RhoA signalling pathway (leading to activation of RhoGEF) as well as to Ca^2+^-handling in ASM [54]. Thus, tyrosine kinase inhibitors could prove valuable in the treatment of asthma, if a sufficiently selective molecule can be found.

### Actomyosin ATPase activity and cross-bridge cycling

Rather than interfering with various "up-stream" signalling events, it could be much more effective to target the penultimate step in excitation-contraction coupling. Activation of actomyosin ATPase activity, through the phosphorylation of myosin light chain, and cross-bridge cycling are the final determinants in the overall cascade of events leading to contraction. A direct inhibitor of MLCK could be far more effective than intervening further upstream using β-agonists and phosphodiesterase inhibitors. On the other hand, MLCP offers a tantalizing target: the identification of a compound which directly, and hopefully selectively, stimulates this activity would be equally effective in the treatment of asthma. In contrast to the extensive literature at hand pertaining to kinases and the availability of innumerable "selective" kinase inhibitors, the phosphatase field is still in its infancy: relatively few selective inhibitors are yet available, perhaps in part because the actual catalytic subunit of these enzymes acts non-selectively on a wide variety of substrates but is brought into proximity of a specific substrate by the targeting subunit. As such, perhaps the targeting subunit should itself be targeted by researchers. Clearly, any putative MLCK inhibitors or MLCP stimulants to be developed for use in asthma would have to contend with the issue of unwanted systemic effects, given the importance of these enzymes in a wide variety of processes and cell types. However, as pointed out above, it may be possible to limit the systemic delivery of any trial compounds by developing them as inhaled agents and/or using gene therapeutic approaches.

The so-called thick filament-mediated mechanisms – those centering around myosin – have eclipsed research in ASM excitation-contraction coupling in large part, and the development of anti-asthma therapies in total. The growing understanding of the importance of thin filament-mediated mechanisms in smooth muscle contraction may eventually reveal other therapeutic approaches for dealing with airway bronchospasm.

### Approaches designed to decrease ASM mass *per se*

A radically different approach would be to ablate the ASM itself, rather than modulate its activity. The question of "why do we have airway smooth muscle" has been raised repeatedly in the past with no convincing and satisfying answers yet (this question is deftly reviewed in ref. [112]). An exciting new development in this arena has been the controlled delivery of thermal energy to the airways using an intrabronchial catheter: a process now referred to as bronchial thermoplasty [113,114]. This technique was originally intended to serve as a treatment for chronic obstructive pulmonary disorder, in which collapse of the airways and gas trapping is a major problem: as such, the thought was that inducing scarring of the airways might make them stiffer and thus remain patent. Instead, no scarring is evident and the airways look completely normal except for the peculiar absence of smooth muscle cells; patients also commented on improved lung function and reduction of symptoms related to asthma. Pre-clinical development-stage work was done in dogs, and included a long series of studies aimed at determining the intensity and duration of delivery of radiofrequency energy required to achieve 50% reduction in ASM mass. The procedure was next tested in a small group of mild asthmatics, and is now being tested in a group of moderate-severe asthmatics. The success of this approach underscores the potential value in developing other means to eradicate the ASM, including the smaller airways. It may be possible to develop toxic chemical interventions which could be delivered specifically to the ASM (*e.g*., via gene therapeutic approaches). Further studies of the cell cycle of ASM are essential, since it may eventually be possible to inhibit ASM proliferation and/or promote ASM apoptosis, both of which would achieve the same desired goal of decreasing overall ASM muscle mass. Likewise, a better understanding of ASM migration could lead to the development of agents which prevent the hypertrophy/hyperplasia which accompany asthma.

### Prospects for the future

As stated above, there have not been any substantially new pharmacological advances in the past decade or two with respect to treatment strategies for asthma which target the ASM. Admittedly, there have been newer β-agonists or phosphodiesterase inhibitors, but these represent only modifications of decades-old strategies. Any truly new advances have been aimed at controlling inflammation, which is also important but should not eclipse any efforts aimed at controlling bronchoconstriction directly. We have stated repeatedly that a better understanding of the mechanisms underlying ASM contraction and AHR is a prerequisite for any such new advances, and that it would be unwise to base any such understanding solely on work being done in the vascular smooth muscle field, let alone others studying non-muscle tissues.

Physiological studies have for too long suffered from important design flaws and limitations. First, the vast majority of studies have been done using tracheal smooth muscle rather than the smaller airways which are far more important in determining resistance to airflow and which are the clinically relevant site of airway inflammation: compounding this shortsightedness is the growing body of literature which shows major structural and functional differences between the large and small airways. Also, too many use maximally effective concentrations of excitatory stimuli – *e.g*., near millimolar concentrations of cholinergic agonists – even though such degrees of stimulation are rarely (if ever) reached in nature; this problem is exacerbated by numerous studies which suggest the relative contributions of various signalling events can vary over the full range of a concentration-response relationship. Mitchell and Sparrow have elegantly shown that only the lower half of the full concentration-response relationship may be relevant, since complete airway closure can occur at roughly the half-maximally effective concentration [115]. As such, any further increase in tension seen at higher concentrations would be completely occult: thus, we need to focus instead on submaximal or even threshold responses. Related to this point, many are now showing that isotonic recordings (in which the muscle shortens as tone develops) capture information which is unavailable or distorted in isometric studies (the mainstay of most studies of ASM physiology and pharmacology). Finally, the bulk of the data pertaining to this matter were obtained under static conditions, whereas very recent work now shows ASM function to be powerfully modulated by mechanical perturbations (stretch; deep inspirations; *etc*.) [116-118]. It is becoming increasingly clear that this is related to a dynamic re-organization of the actin and myosin filaments during contraction. This adaptation of ASM to its microenvironment ('plasticity') may explain many lung/airway phenomena and offer clues for novel therapeutic intervention.

A major and fundamental limitation in studies aimed at better understanding and treating asthma has been the lack of a good animal model of asthma. Asthma is characterized, in part, by AHR, reversible bronchoconstriction, wheezing, inflammation, and cellular changes related to the muscle (hypertrophy and/or hyperplasia), epithelium (denudation; mucous production), and inflammatory cells (infiltration; degranulation; phenotypic changes). There are many animal models which feature a degree of AHR (*e.g*., induced by allergens or noxious agents) which may or may not be accompanied by inflammation, or which reproduce many features of airway inflammation without a change in ASM responsiveness. Regarding those studies which do find AHR in an experimental model, this is usually minor compared to that seen in asthmatics: there is generally only a modest increase in the maximal response and a slight leftward shift, compared to the dramatic shift of several log units in the human condition. Animals do not wheeze (although horses can manifest heaves). In summary, there is no animal model which reproduces fully all the features of asthma. Ultimately, our goal should be to better understand excitation-contraction coupling in human ASM, and changes in that coupling should be studied in tissues from asthmatics.

## Abbreviations

AHR airway hyperresponsiveness

ASM airway smooth muscle

MLCK myosin light chain kinase

MLCP myosin light chain phosphatase

RhoGAP Rho-GTPase activating protein

RhoGDI Rho-specific GDP dissociation inhibitor

RhoGEF Rho-specific guanine nucleotide exchange factor

ROCK Rho-kinase

SERCA sarcoplasmic/endoplasmic reticulum Ca^2+^-ATPase

SRSA slow-reacting substance of anaphylaxis

TRP transient receptor potential

## Competing interests

The author(s) declare that they have no competing interests.

## References

[B1] Fuhlbrigge AL, Adams RJ, Guilbert TW, Grant E, Lozano P, Janson SL, Martinez F, Weiss KB, Weiss ST (2002). The burden of asthma in the United States: level and distribution are dependent on interpretation of the national asthma education and prevention program guidelines. Am J Respir Crit Care Med.

[B2] Fleckenstein A, Frey M, Fleckenstein-Grun G (1986). Antihypertensive and arterial anticalcinotic effects of calcium antagonists. American Journal of Cardiology.

[B3] Faraci FM, Heistad DD (1998). Regulation of the cerebral circulation: role of endothelium and potassium channels. Physiological Reviews.

[B4] Quayle JM, Nelson MT, Standen NB (1997). ATP-sensitive and inwardly rectifying potassium channels in smooth muscle. Physiol Rev.

[B5] Amrani Y, Bronner C (1993). Tumor necrosis factor alpha potentiates the increase in cytosolic free calcium induced by bradykinin in guinea-pig tracheal smooth muscle cells. C R Acad Sci III.

[B6] Amrani Y, Panettieri RA, Frossard N, Bronner C (1996). Activation of the TNF alpha-p55 receptor induces myocyte proliferation and modulates agonist-evoked calcium transients in cultured human tracheal smooth muscle cells. Am J Respir Cell Mol Biol.

[B7] Zacour ME, Tolloczko B, Martin JG (2000). Calcium and growth responses of hyperresponsive airway smooth muscle to different isoforms of platelet-derived growth factor (PDGF). Can J Physiol Pharmacol.

[B8] Bergner A, Sanderson MJ (2002). Acetylcholine-induced calcium signaling and contraction of airway smooth muscle cells in lung slices. J Gen Physiol.

[B9] Bergner A, Sanderson MJ (2002). ATP stimulates Ca^2+ ^oscillations and contraction in airway smooth muscle cells of mouse lung slices. Am J Physiol Lung Cell Mol Physiol.

[B10] Kuo KH, Dai J, Seow CY, Lee CH, van Breemen C (2003). Relationship between asynchronous Ca^2+ ^waves and force development in intact smooth muscle bundles of the porcine trachea. Am J Physiol Lung Cell Mol Physiol.

[B11] Nuttle LC, Farley JM (1996). Frequency modulation of acetylcholine-induced oscillations in Ca^2+ ^and Ca^2+^-activated Cl-current by cAMP in tracheal smooth muscle. J Pharmacol Exp Ther.

[B12] Prakash YS, Pabelick CM, Kannan MS, Sieck GC (2000). Spatial and temporal aspects of ACh-induced [Ca^2+^]_i _oscillations in porcine tracheal smooth muscle. Cell Calcium.

[B13] Roux E, Guibert C, Savineau JP, Marthan R (1997). [Ca^2+^]_i _oscillations induced by muscarinic stimulation in airway smooth muscle cells: receptor subtypes and correlation with the mechanical activity. Br J Pharmacol.

[B14] Ratz PH, Berg KM, Urban NH, Miner AS (2005). Regulation of smooth muscle calcium sensitivity: KCl as a calcium-sensitizing stimulus. Am J Physiol Cell Physiol.

[B15] Liu C, Zuo J, Pertens E, Helli PB, Janssen LJ (2005). Regulation of Rho/ROCK signaling in airway smooth muscle by membrane potential and [Ca^2+^]_i_. Am J Physiol Lung Cell Mol Physiol.

[B16] Morgan KG, Gangopadhyay SS (2001). Invited review: cross-bridge regulation by thin filament-associated proteins. J Appl Physiol.

[B17] Frank DE, Maclaren WR (1957). The use of an anticholinergic drug in the treatment of asthma. Ann Allergy.

[B18] Silbert NE (1952). Treatment of bronchial conditions with a combination of anticholinergic, adrenergic, and antihistaminic drugs; preliminary report. Ann Allergy.

[B19] Brocklehurst WE (1970). The role of slow-reacting substance in asthma. Adv Drug Res.

[B20] Triggle DJ (1983). Calcium, the control of smooth muscle function and bronchial hyperreactivity. Allergy.

[B21] Middleton E (1984). Airway smooth muscle, asthma, and calcium ions. J Allergy Clin Immunol.

[B22] Barnes PJ (1985). Clinical studies with calcium antagonists in asthma. Br J Clin Pharmacol.

[B23] Fish JE (1984). Calcium channel antagonists in the treatment of asthma. J Asthma.

[B24] Gordon EH, Wong SC, Klaustermeyer WB (1987). Comparison of nifedipine with a new calcium channel blocker, flordipine, in exercise-induced asthma. J Asthma.

[B25] Hoppe M, Harman E, Hendeles L (1992). The effect of inhaled gallopamil, a potent calcium channel blocker, on the late-phase response in subjects with allergic asthma. J Allergy Clin Immunol.

[B26] Middleton E (1985). The treatment of asthma – beyond bronchodilators. N Engl Reg Allergy Proc.

[B27] Riska H, Stenius-Aaniala B, arvi AR (1986). Comparison of the efficacy of an ACE-inhibitor and a calcium channel blocker in hypertensive asthmatics. A preliminary report. Postgrad Med J.

[B28] Sly PD, Olinsky A, Landau LI (1986). Does nifedipine affect the diurnal variation of asthma in children?. Pediatr Pulmonol.

[B29] Cook NS, Chapman ID (1993). Therapeutic potential of potassium channel openers in peripheral vascular disease and asthma. Cardiovasc Drugs Ther.

[B30] Faurschou P, Mikkelsen KL, Steffensen I, Franke B (1994). The lack of bronchodilator effect and the short-term safety of cumulative single doses of an inhaled potassium channel opener (bimakalim) in adult patients with mild to moderate bronchial asthma. Pulm Pharmacol.

[B31] Kidney JC, Fuller RW, Worsdell YM, Lavender EA, Chung KF, Barnes PJ (1993). Effect of an oral potassium channel activator, BRL 38227, on airway function and responsiveness in asthmatic patients: comparison with oral salbutamol. Thorax.

[B32] Small RC, Berry JL, Burka JF, SJ Cook, Foster RW, Green KA, Murray MA (1992). Potassium channel activators and bronchial asthma. Clin Exp Allergy.

[B33] Kotlikoff MI, Kamm KE (1996). Molecular mechanisms of beta-adrenergic relaxation of airway smooth muscle. Annu Rev Physiol.

[B34] Janssen LJ, Tazzeo T, Zuo J (2004). Enhanced myosin phosphatase and Ca^2+^-uptake mediate adrenergic relaxation of airway smooth muscle. Am J Respir Cell Mol Biol.

[B35] Liu C, Zuo J, Janssen LJ (2006). Regulation of airway smooth muscle RhoA/ROCK activities by cholinergic and bronchodilator stimuli. Eur Respir J.

[B36] Solway J, Forsythe SM, Halayko AJ, Vieira JE, Hershenson MB, Camoretti-Mercado B (1998). Transcriptional regulation of smooth muscle contractile apparatus expression. Am J Respir Crit Care Med.

[B37] Helli PB, Pertens E, Janssen LJ (2005). Cyclopiazonic acid activates a Ca^2+^-permeable, non-selective cation conductance in porcine and bovine tracheal smooth muscle. J Appl Physiol.

[B38] Li S, Westwick J, Poll C (2003). Transient receptor potential (TRP) channels as potential drug targets in respiratory disease. Cell Calcium.

[B39] Montell C (1997). New light on TRP and TRPL. Mol Pharmacol.

[B40] Gosling M, Poll C, Li S (2005). TRP channels in airway smooth muscle as therapeutic targets. Naunyn Schmiedebergs Arch Pharmacol.

[B41] Ong HL, Barritt GJ (2004). Transient receptor potential and other ion channels as pharmaceutical targets in airway smooth muscle cells. Respirology.

[B42] Bourreau JP, Abela AP, Kwan CY, Daniel EE (1991). Acetylcholine Ca^2+ ^stores refilling directly involves a dihydropyridine-sensitive channel in dog trachea. Am J Physiol.

[B43] Janssen LJ, Sims SM (1993). Emptying and refilling of Ca^2+ ^store in tracheal myocytes as indicated by ACh-evoked currents and contraction. Am J Physiol.

[B44] Qian Y, Bourreau JP (1999). Two distinct pathways for refilling Ca^2+ ^stores in permeabilized bovine trachealis muscle. Life Sci.

[B45] Bourreau JP, Kwan CY, Daniel EE (1993). Distinct pathways to refill ACh-sensitive internal Ca^2+ ^stores in canine airway smooth muscle. Am J Physiol.

[B46] Yang CM, Yo YL, Wang YY (1993). Intracellular calcium in canine cultured tracheal smooth muscle cells is regulated by M_3 _muscarinic receptors. Br J Pharmacol.

[B47] Liu X, Farley JM (1996). Depletion and refilling of acetylcholine- and caffeine-sensitive Ca^2+ ^stores in tracheal myocytes. J Pharmacol Exp Ther.

[B48] Rosado JA, Sage SO (2000). The actin cytoskeleton in store-mediated calcium entry. J Physiol.

[B49] Patterson RL, van Rossum DB, Gill DL (1999). Store-operated Ca^2+ ^entry: evidence for a secretion-like coupling model. Cell.

[B50] Yao Y, Ferrer-Montiel AV, Montal M, Tsien RY (1999). Activation of store-operated Ca^2+ ^current in Xenopus oocytes requires SNAP-25 but not a diffusible messenger. Cell.

[B51] Berridge MJ (1995). Capacitative calcium entry. Biochem J.

[B52] Kobayashi S, Gong MC, Somlyo AV, Somlyo AP (1991). Ca^2+ ^channel blockers distinguish between G protein-coupled pharmacomechanical Ca^2+ ^release and Ca^2+ ^sensitization. Am J Physiol.

[B53] Rosales C, Brown EJ (1992). Calcium channel blockers nifedipine and diltiazem inhibit Ca^2+ ^release from intracellular stores in neutrophils. J Biol Chem.

[B54] Tolloczko B, Tao FC, Zacour ME, Martin JG (2000). Tyrosine kinase-dependent calcium signaling in airway smooth muscle cells. Am J Physiol Lung Cell Mol Physiol.

[B55] Small RC, Boyle JP, Duty S, Elliott KR, Foster RW, Watt AJ (1989). Analysis of the relaxant effects of AH 21–132 in guinea-pig isolated trachealis. Br J Pharmacol.

[B56] Smith PG, Garcia R, Kogerman L (1998). Mechanical strain increases protein tyrosine phosphorylation in airway smooth muscle cells. Exp Cell Res.

[B57] Emala CW, Liu F, Hirshman CA (1999). Gialpha but not gqalpha is linked to activation of p21(ras) in human airway smooth muscle cells. Am J Physiol.

[B58] Hakonarson H, Grunstein MM (1998). Regulation of second messengers associated with airway smooth muscle contraction and relaxation. Am J Respir Crit Care Med.

[B59] Hirshman CA, Emala CW (1999). Actin reorganization in airway smooth muscle cells involves G_q _and G_i-2 _activation of Rho. Am J Physiol.

[B60] Togashi H, Emala CW, Hall IP, Hirshman CA (1998). Carbachol-induced actin reorganization involves G_i _activation of Rho in human airway smooth muscle cells. Am J Physiol.

[B61] Gunst SJ, Tang DD (2000). The contractile apparatus and mechanical properties of airway smooth muscle. Eur Respir J.

[B62] Madison JM, Ethier MF, Yamaguchi H (1998). Refilling of caffeine-sensitive intracellular calcium stores in bovine airway smooth muscle cells. Am J Physiol.

[B63] Tang DD, Gunst SJ (2001). Depletion of focal adhesion kinase by antisense depresses contractile activation of smooth muscle. Am J Physiol Cell Physiol.

[B64] Perez JF, Sanderson MJ (2005). The contraction of smooth muscle cells of intrapulmonary arterioles is determined by the frequency of Ca^2+ ^oscillations induced by 5-HT and KCl. J Gen Physiol.

[B65] Perez JF, Sanderson MJ (2005). The frequency of calcium oscillations induced by 5-HT, Ach and KCl determine the contraction of smooth muscle cells of intrapulmonary bronchioles. J Gen Physiol.

[B66] Janssen LJ, Sims SM (1995). Ca^2+^-dependent Cl-current in canine tracheal smooth muscle cells. Am J Physiol.

[B67] ZhuGe R, Sims SM, Tuft RA, Fogarty KE, Walsh JV (1998). Ca^2+ ^sparks activate K^+ ^and Cl-channels, resulting in spontaneous transient currents in guinea-pig tracheal myocytes. J Physiol.

[B68] Wang YX, Kotlikoff MI (1997). Inactivation of calcium-activated chloride channels in smooth muscle by calcium/calmodulin-dependent protein kinase. Proc Natl Acad Sci USA.

[B69] Waniishi Y, Inoue R, Morita H, Teramoto N, Abe K, Ito Y (1998). Cyclic GMP-dependent but G-kinase-independent inhibition of Ca^2+^-dependent Cl-currents by NO donors in cat tracheal smooth muscle. J Physiol.

[B70] Janssen LJ, Sims SM (1992). Acetylcholine activates non-selective cation and chloride conductances in canine and guinea-pig tracheal myocytes. J Physiol.

[B71] Janssen LJ, Sims SM (1993). Histamine activates Cl- and K^+ ^currents in guinea-pig tracheal myocytes: convergence with muscarinic signalling pathway. J Physiol.

[B72] Oonuma H, Nakajima T, Nagata T, Iwasawa K, Wang Y, Hazama H, Morita Y, Yamamoto K, Nagai R, Omata M (2000). Endothelin-1 is a potent activator of nonselective cation currents in human bronchial smooth muscle cells. Am J Respir Cell Mol Biol.

[B73] Ahern GP, Laver DR (1998). ATP inhibition and rectification of a Ca^2+^-activated anion channel in sarcoplasmic reticulum of skeletal muscle. Biophys J.

[B74] Janssen LJ (2002). Ionic mechanisms and Ca^2+ ^regulation in airway smooth muscle contraction: do the data contradict dogma?. Am J Physiol Lung Cell Mol Physiol.

[B75] Hirota S, Trimble N, Pertens E, Janssen LJ (2006). Intracellular Cl-fluxes play a novel role in Ca^2+^-handling in airway smooth muscle. Am J Physiol Lung Cell Mol Physiol.

[B76] Higashijima T, Ferguson KM, Sternweis PC (1987). Regulation of hormone-sensitive GTP-dependent regulatory proteins by chloride. J Biol Chem.

[B77] Iizuka K, Yoshii A, Samizo K, Tsukagoshi H, Ishizuka T, Dobashi K, Nakazawa T, Mori M (1999). A major role for the rho-associated coiled coil forming protein kinase in G-protein-mediated Ca^2+ ^sensitization through inhibition of myosin phosphatase in rabbit trachea. Br J Pharmacol.

[B78] Nakahara T, Moriuchi H, Yunoki M, Sakamato K, Ishii K (2000). Y-27632 potentiates relaxant effects of beta 2-adrenoceptor agonists in bovine tracheal smooth muscle. Eur J Pharmacol.

[B79] Yoshii A, Iizuka K, Dobashi K, Horie T, Harada T, Nakazawa T, Mori M (1999). Relaxation of contracted rabbit tracheal and human bronchial smooth muscle by Y-27632 through inhibition of Ca^2+ ^sensitization. Am J Respir Cell Mol Biol.

[B80] Hashimoto T, Nakano Y, Yamashita M, Fang YI, Ohata H, Momose K (2002). Role of Rho-associated protein kinase and histamine in lysophosphatidic acid-induced airway hyperresponsiveness in guinea pigs. Jpn J Pharmacol.

[B81] Chiba Y, Takada Y, Sakai H, Takeyama H, Misawa M (2000). Acetylcholine-induced smooth muscle contraction of intrapulmonary small bronchi is augmented in antigen-induced airway hyperresponsive rats. Jpn J Pharmacol.

[B82] Chiba Y, Takada Y, Miyamoto S, MitsuiSaito M, Karaki H, Misawa M (1999). Augmented acetylcholine-induced, Rho-mediated Ca^2+ ^sensitization of bronchial smooth muscle contraction in antigen-induced airway hyperresponsive rats. Br J Pharmacol.

[B83] Chiba Y, Sakai H, Misawa M (2001). Augmented acetylcholine-induced translocation of RhoA in bronchial smooth muscle from antigen-induced airway hyperresponsive rats. Br J Pharmacol.

[B84] Chiba Y, Misawa M (2004). The role of RhoA-mediated Ca^2+ ^sensitization of bronchial smooth muscle contraction in airway hyperresponsiveness. J Smooth Muscle Res.

[B85] Chiba Y, Sakai H, Wachi H, Sugitani H, Seyama Y, Misawa M (2003). Upregulation of RhoA mRNA in bronchial smooth muscle of antigen-induced airway hyperresponsive rats. J Smooth Muscle Res.

[B86] Sakai H, Otogoto S, Chiba Y, Abe K, Misawa M (2004). TNF-alpha augments the expression of RhoA in the rat bronchus. J Smooth Muscle Res.

[B87] Croxton TL, Lande B, Hirshman CA (1998). Role of G proteins in agonist-induced Ca^2+ ^sensitization of tracheal smooth muscle. Am J Physiol.

[B88] Janssen LJ, Tazzeo T, Zuo J, Pertens E, Keshavjee S (2004). KCl evokes contraction of airway smooth muscle via activation of RhoA and Rho-kinase. Am J Physiol Lung Cell Mol Physiol.

[B89] Janssen LJ, Wattie J, Lu-Chao H, Tazzeo T (2001). Muscarinic excitation-contraction coupling mechanisms in tracheal and bronchial smooth muscles. J Appl Physiol.

[B90] Rosenfeldt HM, Amrani Y, Watterson KR, Murthy KS, Panettieri RA, Spiegel S (2003). Sphingosine-1-phosphate stimulates contraction of human airway smooth muscle cells. FASEB J.

[B91] Kobrinsky E, Schwartz E, Abernethy DR, Soldatov NM (2003). Voltage-gated mobility of the Ca^2+ ^channel cytoplasmic tails and its regulatory role. J Biol Chem.

[B92] Soldatov NM (2003). Ca^2+ ^channel moving tail: link between Ca^2+^-induced inactivation and Ca^2+ ^signal transduction. Trends Pharmacol Sci.

[B93] Kimura K, Ito M, Amano M, Chihara K, Fukata Y, Nakafuku M, Yamamori B, Feng J, Nakano T, Okawa K, Iwamatsu A, Kaibuchi K (1996). Regulation of myosin phosphatase by Rho and Rho-associated kinase (Rho-kinase). Science.

[B94] Kosako H, Yoshida T, Matsumura F, Ishizaki T, Narumiya S, Inagaki M (2000). Rho-kinase/ROCK is involved in cytokinesis through the phosphorylation of myosin light chain and not ezrin/radixin/moesin proteins at the cleavage furrow. Oncogene.

[B95] Yamashiro S, Totsukawa G, Yamakita Y, Sasaki Y, Madaule P, Ishizaki T, Narumiya S, Matsumura F (2003). Citron kinase, a Rho-dependent kinase, induces di-phosphorylation of regulatory light chain of myosin II. Mol Biol Cell.

[B96] Matsui T, Maeda M, Doi Y, Yonemura S, Amano M, Kaibuchi K, Tsukita S, Tsukita S (1998). Rho-kinase phosphorylates COOH-terminal threonines of ezrin/radixin/moesin (ERM) proteins and regulates their head-to-tail association. J Cell Biol.

[B97] Fukata Y, Kimura K, Oshiro N, Saya H, Matsuura Y, Kaibuchi K (1998). Association of the myosin-binding subunit of myosin phosphatase and moesin: dual regulation of moesin phosphorylation by Rho-associated kinase and myosin phosphatase. J Cell Biol.

[B98] Oshiro N, Fukata Y, Kaibuchi K (1998). Phosphorylation of moesin by rho-associated kinase (Rho-kinase) plays a crucial role in the formation of microvilli-like structures. J Biol Chem.

[B99] Fukata Y, Oshiro N, Kaibuchi K (1999). Activation of moesin and adducin by Rho-kinase downstream of Rho. Biophys Chem.

[B100] Izawa T, Fukata Y, Kimura T, Iwamatsu A, Dohi K, Kaibuchi K (2000). Elongation factor-1 alpha is a novel substrate of rho-associated kinase. Biochem Biophys Res Commun.

[B101] Fukata Y, Oshiro N, Kinoshita N, Kawano Y, Matsuoka Y, Bennett V, Matsuura Y, Kaibuchi K (1999). Phosphorylation of adducin by Rho-kinase plays a crucial role in cell motility. J Cell Biol.

[B102] Goto H, Kosako H, Inagaki M (2000). Regulation of intermediate filament organization during cytokinesis: possible roles of Rho-associated kinase. Microsc Res Tech.

[B103] Kosako H, Goto H, Yanagida M, Matsuzawa K, Fujita M, Tomono Y, Okigaki T, Odai H, Kaibuchi K, Inagaki M (1999). Specific accumulation of Rho-associated kinase at the cleavage furrow during cytokinesis: cleavage furrow-specific phosphorylation of intermediate filaments. Oncogene.

[B104] Goto H, Kosako H, Tanabe K, Yanagida M, Sakurai M, Amano M, Kaibuchi K, Inagaki M (1998). Phosphorylation of vimentin by Rho-associated kinase at a unique amino-terminal site that is specifically phosphorylated during cytokinesis. J Biol Chem.

[B105] Maekawa M, Ishizaki T, Boku S, Watanabe N, Fujita A, Iwamatsu A, Obinata T, Ohashi K, Mizuno K, Narumiya S (1999). Signaling from Rho to the actin cytoskeleton through protein kinases ROCK and LIM-kinase. Science.

[B106] Ohashi K, Nagata K, Maekawa M, Ishizaki T, Narumiya S, Mizuno K (2000). Rho-associated kinase ROCK activates LIM-kinase 1 by phosphorylation at threonine 508 within the activation loop. J Biol Chem.

[B107] Landmesser U, Bahlmann F, Mueller M, Spiekermann S, Kirchhoff N, Schulz S, Manes C, Fischer D, de Groot K, Fliser D, Fauler G, Marz W, Drexler H (2005). Simvastatin versus ezetimibe: pleiotropic and lipid-lowering effects on endothelial function in humans. Circulation.

[B108] Wassmann S, Faul A, Hennen B, Scheller B, Bohm M, Nickenig G (2003). Rapid effect of 3-hydroxy-3-methylglutaryl coenzyme a reductase inhibition on coronary endothelial function. Circ Res.

[B109] Gerthoffer WT, Gunst SJ (2001). Focal adhesion and small heat shock proteins in the regulation of actin remodeling and contractility in smooth muscle. J Appl Physiol.

[B110] Mehta D, Tang DD, Wu MF, Atkinson S, Gunst SJ (2000). Role of Rho in Ca^2+^-insensitive contraction and paxillin tyrosine phosphorylation in smooth muscle. Am J Physiol Cell Physiol.

[B111] Tang DD, Gunst SJ (2001). Roles of focal adhesion kinase and paxillin in the mechanosensitive regulation of myosin phosphorylation in smooth muscle. J Appl Physiol.

[B112] Mitzner W (2004). Airway smooth muscle: the appendix of the lung. Am J Respir Crit Care Med.

[B113] Cox G, Miller JD, McWilliams A, Fitzgerald JM, Lam S (2006). Bronchial thermoplasty for asthma. Am J Respir Crit Care Med.

[B114] Miller JD, Cox G, Vincic L, Lombard CM, Loomas BE, Danek CJ (2005). A prospective feasibility study of bronchial thermoplasty in the human airway. Chest.

[B115] Mitchell HW, Sparrow MP (1989). The relevance of pharmacological dose – response curves to airway narrowing. Trends Pharmacol Sci.

[B116] Gump A, Haughney L, Fredberg J (2001). Relaxation of activated airway smooth muscle: relative potency of isoproterenol vs. tidal stretch. J Appl Physiol.

[B117] Kuo KH, Wang L, Pare PD, Ford LE, Seow CY (2001). Myosin thick filament lability induced by mechanical strain in airway smooth muscle. J Appl Physiol.

[B118] Waters CM, Sporn PH, Liu M, Fredberg JJ (2002). Cellular biomechanics in the lung. Am J Physiol Lung Cell Mol Physiol.

